# Mutational Bias and Translational Selection Shaping the Codon Usage Pattern of Tissue-Specific Genes in Rice

**DOI:** 10.1371/journal.pone.0048295

**Published:** 2012-10-29

**Authors:** Qingpo Liu

**Affiliations:** College of Agriculture and Food Science, Zhejiang A & F University, Lin’an, People’s Republic of China; University of Lausanne, Switzerland

## Abstract

The regulatory mechanisms of determining which genes specifically expressed in which tissues are still not fully elucidated, especially in plants. Using internal correspondence analysis, I first establish that tissue-specific genes exhibit significantly different synonymous codon usage in rice, although this effect is weak. The variability of synonymous codon usage between tissues accounts for 5.62% of the total codon usage variability, which has mainly arisen from the neutral evolutionary forces, such as GC content variation among tissues. Moreover, tissue-specific genes are under differential selective constraints, inferring that natural selection also contributes to the codon usage divergence between tissues. These findings may add further evidence in understanding the differentiation and regulation of tissue-specific gene products in plants.

## Introduction

Within a genome, some synonymous codons are avoided readily, while some are used preferentially. Such biases are ubiquitously present in eukaryotes and prokaryotes. The processes of shaping codon usage in a given genome are very complicated, which may be primarily attributed to natural selection and neutral evolutionary forces, such as biased mutation and gene conversion. In most cases, codon usage can reflect a balance between natural selection and neutral evolution [1]. However, in some multicellular species, translational selection serves as the major determinant in shaping codon usage [2]–[3]. In contrast, in mammals, especially in human, the synonymous codon usage is mainly arisen from neutral processes rather than selection [4].

Interestingly, significant differences in synonymous codon usage were observed between genes selectively expressed in six adult human tissues; this tissue-specific variation of synonymous codon usage is probably not only due to translational selection but isochore structures [5]. Sémon et al. [6] confirmed the significant (but weak) difference of tissue-specific codon usage; however, they found no evidence for tissue-specific adaptation of synonymous codon usage in human [6]. In addition, it was reported that in plants and mammalians tissue-specific genes are under weak selective constraints, and evolve more quickly than housekeeping genes [7]–[8]. Thus, different evolutionary forces must underline the evolution of synonymous codon usage of housekeeping and tissue-specific genes.

To our knowledge, whether there exists significant codon usage variation and to what extent of these differences between tissue-specifically expressed genes in plants have not been explored yet. Rice is very heterogeneous in base composition, and has an isochore structure similar to mammals [9]. It is thus intriguing to understand how about the codon usage of tissue-specific genes in rice. Particularly, although base compositional mutation bias and natural selection were found to have played crucial roles in determining the codon usage of all rice genes [10], whether the tissue-specific codon usage was affected by both factors remains unclear.

In this study, the tissue-specific codon usage in rice was evaluated using a multivariate method, internal correspondence analysis [11]. These analyses reveal a significant difference in synonymous codon usage between genes selectively expressed in different tissues. As observed in the human genome, however, this effect is weak, which represents only 5.62% of the total codon usage variability. Importantly, the GC-content variation between tissues primarily contributes to, and translational selection may play a relative weaker role in shaping the tissue-specific synonymous codon usage variability in rice.

## Materials and Methods

### Sequence Data

The rice (*Oryza sativa*) protein-coding sequences (CDSs) and expressed sequence tags (ESTs) were retrieved from the rice genomic resource (MSU pseudomolecule v7.0; ftp://ftp.plantbiology.msu.edu/pub/data/Eukaryotic_Projects/o_sativa/annotation_dbs/pseudomolecules) and GenBank database (release 189, April 2012), respectively. If there were several alternative splicing variants, the longest CDS was chosen as the representative for that gene. The total dataset contains 56,591 rice CDSs.

**Table 1:** Comparison of ENC, CAI, and CDS length of tissue- and non-tissue-specific genes in rice.

Tissue	No.Seqs	ENC	CAI	CDSlength
Root	49	40.436±1.419 cd	0.715±0.018 ab	1057.3±89.1 c
Shoot	33	43.340±1.639 c	0.677±0.023 bc	1125.1±118.0 bc
Leaf	356	47.975±0.434 b	0.620±0.006 de	1369.3±63.7 bc
Anther	108	43.268±0.955 c	0.694±0.012 b	1248.1±73.1 bc
Embryo	29	39.615±1.821 d	0.739±0.023 a	1176.6±204.1 bc
Endosperm	36	48.060±1.291 b	0.635±0.019 cd	1533.4±429.4 b
5d-seed	64	51.718±0.864 ab	0.565±0.012 ef	1654.4±149.8 b
Non-specific	1187	53.636±0.127 a	0.538±0.002 f	2455.5±28.8 a

*Note*: Data are reported as means ± SD. Within a column, mean values followed by different letters (a, b, c, d, e, and f) mean significant difference at the 0.05 level (*p*<0.05).

Gene expression patterns were estimated using EST data. A total of 1,252,989 rice ESTs were downloaded, in which ESTs belonging to cDNA libraries from suspension cell culture, callus, pooled or mix organs, or unidentified tissues were excluded. CDSs were then used as query to search against the EST data using MEGABLAST [12] with an E-value cutoff 10^−20^. A sequence match is counted if MEGABLAST alignment shows at least 95% identity over 300 nt or more.

**Figure 1:**
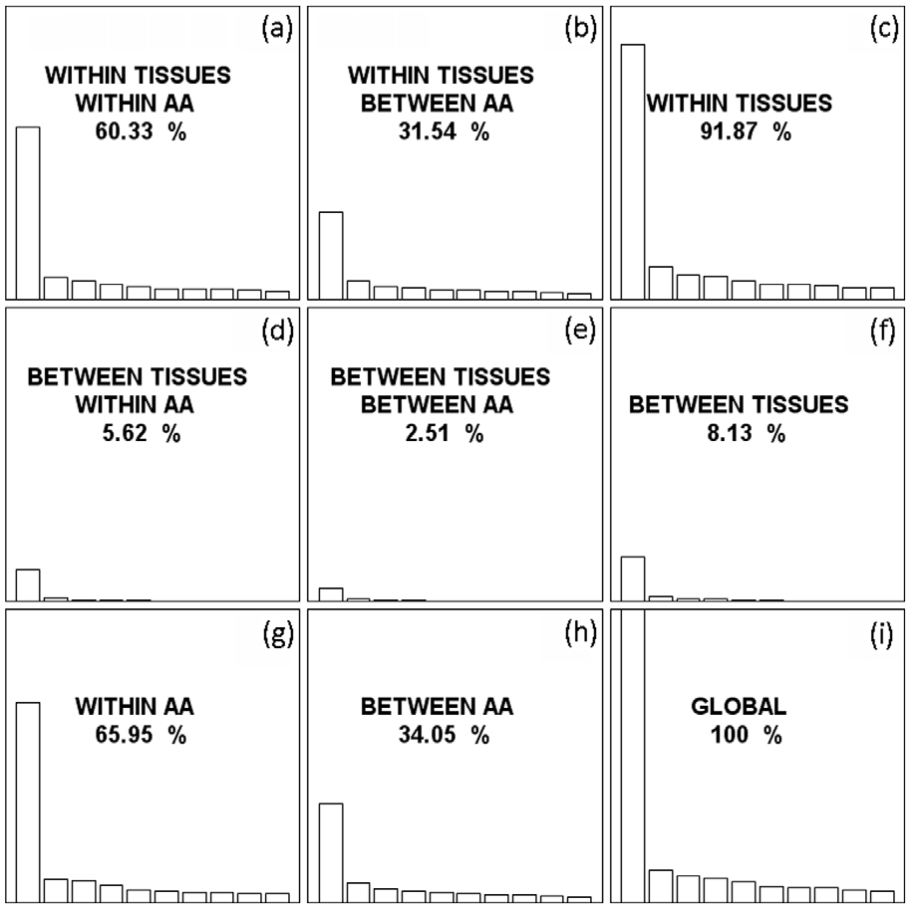
Internal correspondence analysis of tissue-specific genes in rice. The global codon usage variability was decomposed into within-block and between-block variability, consisting of the amino acid usage (between-AA) variability (*b*, *e*, *h*), synonymous codon usage (within-AA) variability (*a*, *d*, *g*), and variability of between- (*d*, *e*, *f*) or within tissues (*a*, *b*, *c*). The decomposition of global variability yields nine elementary analyses (*a*-*i*). In each peculiar analysis, the contribution to the total variance is indicated, where only the first 10 eigenvalues are represented to allow for a direct visual comparison.

The tissue expression pattern of rice genes was also examined by analyzing the published micro-array data (GSE7951, and GSE11966; [13]–[14]), where the gene expression profiles in nine and five rice tissues were included, respectively. The PaGeFinder web-server, an updated version of GEPS [15], was employed to quantitatively analyze the expression pattern of CDSs. Specificity Measure (SPM) was used to define the tissue-specific expression pattern of a gene. According to the definition, if a gene has a SPM value >0.9 in one tissue, it was regarded as a candidate that was specifically expressed in that tissue [15]. Taken together, for this study, a gene was considered to be tissue specific if it has a high SPM value (>0.9) and its transcript is detected in only one rice tissue. Finally a dataset containing 675 tissue-specific genes from seven tissues including root, shoot, leaf, anther, embryo, endosperm, and 5d-seed were used for further analysis.

**Table 2:** Base composition of coding sequences of tissue- and non-tissue-specific genes in rice.

Tissue	No. Seqs	GC1	GC2	GC3	GC all
Root	49	0.590±0.010 ab	0.483±0.013 a	0.733±0.030 a	0.602±0.014 a
Shoot	33	0.595±0.015 a	0.472±0.014 ab	0.708±0.040 a	0.592±0.016 a
Leaf	356	0.555±0.005 cd	0.435±0.005 cd	0.573±0.010 b	0.521±0.006 b
Anther	108	0.582±0.007 ab	0.442±0.007 cd	0.717±0.019 a	0.580±0.010 a
Embryo	29	0.581±0.014 ab	0.450±0.016 bc	0.696±0.033 a	0.576±0.018 a
Endosperm	36	0.567±0.013 bc	0.404±0.012 e	0.550±0.030 bc	0.507±0.017 bc
5d-seed	64	0.531±0.008 d	0.401±0.008 e	0.511±0.019 cd	0.481±0.010 c
Non-specific	1187	0.547±0.001 cd	0.424±0.001 de	0.466±0.003 d	0.479±0.002 c
**Tissue**	**No. Seqs**	**A**	**T**	**C**	**G**
Root	49	0.210±0.002 f	0.188±0.001 g	0.293±0.002 b	0.309±0.001 a
Shoot	33	0.207±0.003 g	0.201±0.002 e	0.297±0.003 a	0.295±0.003 b
Leaf	356	0.248±0.000 c	0.231±0.000 c	0.246±0.000 e	0.275±0.000 c
Anther	108	0.225±0.002 e	0.195±0.001 f	0.284±0.001 c	0.296±0.001 b
Embryo	29	0.239±0.003 d	0.185±0.002 h	0.280±0.003 d	0.296±0.002 b
Endosperm	36	0.270±0.004 a	0.223±0.002 d	0.230±0.004 f	0.276±0.002 c
5d-seed	64	0.266±0.001 b	0.254±0.001 a	0.212±0.001 h	0.269±0.002 d
Non-specific	1187	0.271±0.000 a	0.250±0.000 b	0.224±0.000 g	0.255±0.000 e

*Note*: Data are reported as means ± SD. Within a column, mean values followed by different letters (a, b, c, d, e, f, and g) mean significant difference at the 0.05 level (*p*<0.05).

### Measures of Synonymous Codon Usage Bias

The effective number of codons (ENC), the most common measure of codon bias, was calculated, which yields values ranging from 20 to 61. A higher ENC value means a weaker codon usage bias [16]. The codon adaptation index (CAI), a commonly used measure of the extent of bias toward codons that were known to be preferred in highly expressed genes, was calculated to assess the expression level of tissue-specific genes. A CAI value is between 0 and 1.0, and a higher CAI value means a stronger codon usage bias and a higher expression level [17]. Herein, a set of 71 highly expressed genes plus the ribosomal protein-coding genes were used as reference to calculate CAI values [18] (see Table S1). In addition, the frequency of G + C at the first, second, and third codon position (GC1, GC2, and GC3), and that of the entire gene (GCall) were calculated after excluding the tryptophan, methionine, and three stop codons.

**Figure 2:**
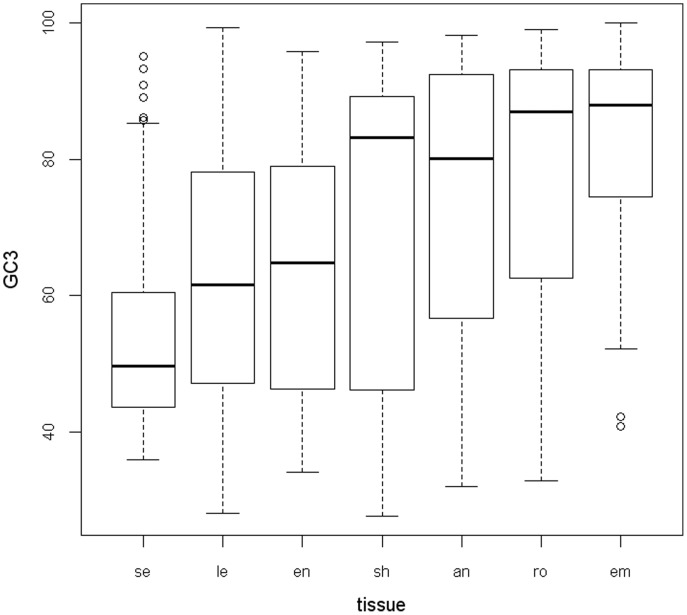
Illustration of GC3 content variation for genes selectively expressed in different tissues. For each tissue, the distribution of GC3 content is represented by a boxplot, where the lower, middle, and top horizontal lines of the boxes represent the 25%, 50%, and 75% quantiles, respectively. Initials of tissue names are indicated: *se*, seed; *le*, leaf; *en*, endosperm; *sh*, shoot; *an*, anther; *ro*, root; *em*, embryo.

**Figure 3:**
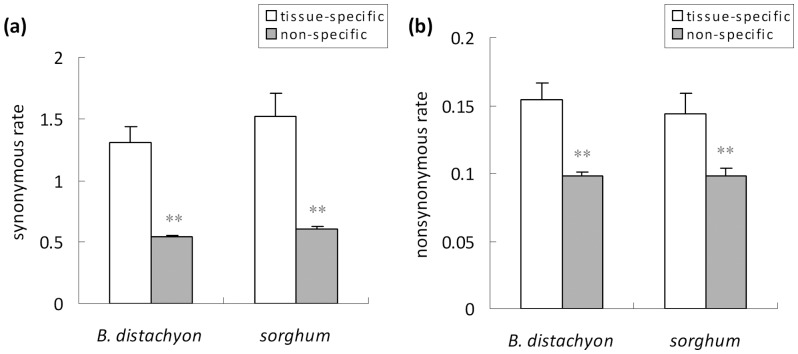
Comparison of synonymous *d*_S_ (a) and nonsynonymous substitution rates *d*_N_ (b) between rice and *B. distachyon*, and *sorghum* orthologous gene pairs for tissue-specific and non-tissue-specific classes of genes. The symbol ** indicates significant difference at the 0.01 level (*p*<0.01).

### Internal Correspondence Analysis

The seqinr [19] and ade4 [20] packages implemented in R [21] were employed to perform the internal correspondence analysis (ICA), which is an extension of correspondence analysis [18] and has been applied in several codon usage studies [6], [22]. Briefly, a codon usage table consisting of 675 rows, each corresponding to a CDS, and 61 columns corresponding to the 61 genetic codons was constructed and further used to investigate the inter- and intra-tissue variability. According to ICA, the rows and columns can be split into 7 and 20 blocks that are corresponding to the different tissues and different amino acids, respectively. Based on this, the total codon usage variability can be further decomposed into between-block and within-block variability. Therefore, it is likely to infer which part of the total codon usage variability is due to variability between different tissues [6], [22].

### Statistic Analysis

The indices of measure of synonymous codon usage bias, including ENC, CAI, GCall, GC1, GC2, GC3, and CDS length, were calculated using CodonW v1.4 [23] and custom PERL programs, respectively. The internal correspondence analysis [20], *Spearman* correlation, and ANOVA analysis were all performed using the R software v2.15 [21].

### Identification of Orthologous Gene Pairs

The amino acids, CDS, and EST sequences of *Sorghum bicolor* and *Brachypodium distachyon* were downloaded from the Phytozome (version 8.0; http://www.phytozome.net/) and GenBank databases (release 189, April 2012), respectively. Totally, 208,841 and 128,092 *S. bicolor* and *B. distachyon* ESTs were downloaded, which were then rearranged as that described in rice. The orthologous gene pairs of rice, and *S. bicolor*, and *B. distachyon* were obtained from the PhylomeDB (v3.0; [24]) and MetaPhOrs [25] databases, where the orthologs were identified using the phylogeny-based approaches [24]–[26] instead of simple BLAST search [27]. The tissue- and non-tissue-specific genes identified in rice were used as query to search against the *S. bicolor* and *B. distachyon* sequences to identify their orthologous counterparts in each of the two species, using a custom PERL program. In order to efficiently distinguish the tissue-specific and non-tissue-specific genes in *S. bicolor* and *B. distachyon*, the ESTs originated from rice and the two plant species were mixed together to construct an EST sequences pool, considering that the three species are phylogenetically close to each other. After that, the identified orthologous genes in *S. bicolor* and *B. distachyon* were separately searched against the EST sequences pool using MEGABLAST [12]. The tissue expression pattern of a given gene was inferred based on their EST profiling. Here, if its transcript is detected in only one tissue, the corresponding gene was referred to be tissue-specific. On the contrary, a gene was regarded to be non-tissue-specific, if it was expressed in two tissues or more but not significantly abundance in any tissues. The amino acid sequences of orthologs were aligned using MAFFT v6.6 [28], and then the codon-alignments of CDS sequences were generated based on the resulting amino acid alignments using a custom PERL program. The Yang and Nielsen method [29] implemented in the yn00 program of the PAML v4.4 package [30] was employed to calculate the pair-wise synonymous (*d*_S_) and non-synonymous (*d*_N_) distance between the orthologous genes of rice and *S. bicolor* and *B. distachyon*.

## Results and Discussion

### Variation in Synonymous Codon Usage between Tissues

The data set consisting of 675 CDSs from seven tissues were subjected to CodonW and custom PERL programs to investigate the synonymous codon usage of rice tissues. From Table 1, it is obvious that the average ENC value in one tissue is significantly different from the ones in other tissues. The similar trend can be observed by analyzing the CAI values, another commonly used measure of codon bias. In addition, ENC values are significantly negatively correlated with CAI values (Spearman’s correlation coefficient *r* = −0.964, *p* = 0.003), a result that is consistent with those in analyzing all rice genes [10]. It is worthy to note that the average ENC and CAI values of genes selectively expressed in 5d-seed are significantly greater and smaller than that of other tissues, indicating that 5d-seed-tissue-specific genes may tend to select synonymous codons randomly. The reverse case was found in embryo, where the average ENC value is significantly smaller than other tissues, indicative of stronger codon usage bias. These observations suggest that remarkable variation in synonymous codon usage should have occurred between different tissues in rice. Compared with the genes that are tissue-specific, non-tissue-specific genes have the weakest codon usage bias, and are usually expressed at a lower level (Table 1).

However, ENC only measures the overall departure from random synonymous codon choice, and hence which codons are used more frequent than others can not be clearly presented. As a result, two genes exhibiting the same degree of overall bias (ENC value) may actually differ dramatically in their particular choice of synonymous codons [5]. To cope with this problem, Plotkin et al. [5] developed a new method to measure the distance between two genes by counting the number of amino acids that exhibit significantly different codon usage. However, this method is also defective because it is sensitive to the length and the amino acid composition of proteins [6]. Thus, for this study, the internal correspondence analysis (ICA) was adopted to further investigate the variability of synonymous codon usage among tissue-specific genes in different tissues.

In ICA, the total codon usage variability can be decomposed into four parts of codon usage variability, including the amino acid usage variability (between-AA variability), synonymous codon usage variability (within-AA variability), and variability between or within different tissues. The performance of ICA yields nine elementary analyses (Fig. 1). It was observed that 91.87% and 8.13% of total codon usage variability are due to variability within and between different tissues, respectively (Figs. 1*c and 1f*), suggesting that the observed differences in synonymous codon usage (Table 1) may mainly arise from the differences within different tissues. On the other hand, 65.95% and 34.05% of global variability are due to variability in synonymous codon usage (within-AA; Fig. 1*g*) and to variability in amino acid usage (between-AA; Fig. 1*h*), respectively. However, the effect of variability in synonymous codon usage between tissues is much small as compared to other sources of variability, which only accounts for 5.62% of the total variability in codon usage (Fig. 1*d*).

In order to test whether the observed small proportion of variability in synonymous codon usage between tissues (Fig. 1*d*) is caused just by chance, 1,000 independent permutations were performed by randomly associating the genes and tissues, and the ICA was repeated accordingly. It was found that the observed value (5.62%) is significantly greater than that obtained by chance (0.715±0.008%, *p*<0.001), indicating that tissue-specific genes are indeed significantly different in synonymous codon usage that depends on the tissue where they are expressed.

As reported, the identification of tissue-specific genes is always determinant with the sensitivity of the method employed to detect expression [6]. To test this hypothesis, two groups of new datasets were presented. One is composed of genes that are only detected in one of the seven tissues by EST profiling. After running the ICA on this new dataset, I found that the fraction of variability of synonymous codon usage between tissues decreases up to 3.6%. Alternatively, if the tissue specificity was defined based on the definition proposed by Wang et al. [15], only 0.8% of total variability could be attributed to the variation in synonymous codon usage between tissues. However, using a combination of the two methods, this fraction of value climbs up to 5.62%, which is nearly 2.5 times of that reported in human (2.3%, [6]), where the tissue-specific genes were defined only based on the EST abundance of transcripts. As known, EST sequences are often used to estimate the expression profiling of protein-coding genes. However, the sensitivity of this method always depends on cDNA libraries’ origin, EST data quality as well as the numbers of EST sequences in each library, which can greatly influence the final annotation of tissue-specific genes [31]. In addition, if a gene has a SPM value close to 1, as proposed by Wang et al. [15], the corresponding gene can be designated as tissue-specific. According to the definition, the SPM value >0.9 is used as empirical cutoff in classifying tissue-specific genes [15]. In this regard, this method is also defective, because in some cases genes that are expressed at particularly high levels in a given tissue but also expressed at relatively lower levels in other tissues are also considered as tissue-specific. In this study, the tissue-specific genes identified by the two methods were integrated effectively, thus the obtained results should be more robust than simply adopting either EST profiling or Specificity Measure (SPM).

### Base Composition and Tissue-specific Synonymous Codon Usage

The nucleotide compositions of coding sequences of seven tissues were shown in Table 2. In the analysis of mononucleotide composition, it is obvious that the A, T, C, and G contents are clearly differential between different tissues. For example, genes selectively expressed in 5d-seed and leaf contain higher A and T, whereas genes in the root and shoot tissues tend to use C and G frequently. In addition, the global GC content (GCall) and the percent of GC for the three codon positions were all clearly higher in root and shoot than in other tissues. Differences in GC content are largest at the third codon position (GC3), and followed by the second (GC2) and first (GC1) codon positions. In non-tissue-specific genes, the first and second codon positions (GC1 and GC2) have the highest and lowest GC content, respectively. Notably, non-tissue-specific genes has the lowest average GC content (GCall) compared with tissue-specific genes, although its GCall value is not significantly different from those of 5d-seed- and endosperm-specific genes (Table 2).

To test whether the observed significant difference in synonymous codon usage between tissues can be solely explained by the differences in base compositions, the ICA analysis of within-AA and between-tissues variability in codon usage (Fig. 1*d*) was analyzed, where a single major trend in codon usage was identified: the first axis may account for 83.1% of the total variation in synonymous codon usage between tissues. Thus, the first axis can be used as the major factor explaining the variation in codon usage between tissues. It was observed that Axis 1 coordinates are strongly negatively correlated with GC, and GC3 content (Spearman’s correlation coefficients *r* = −0.964, *p* = 0.0028; and *r* = −0.929, *p* = 0.0067, respectively). Particularly, the GC3 content of tissue-specific genes varies extensively, and the variation of GC3 content between genes within a given tissue is even much larger than the variability between tissues (Fig. 2). Thus, the weak differences of GC content between genes specifically expressed in different tissues mainly contribute to the observed differences of synonymous codon usage between tissues.

In addition, a significantly positive *Spearman* correlation between Axis 1 coordinates and intronic GC content was found (*r* = 0.955, *p* = 0.0008). Under mutational bias alone, the correlations between Axis 1 and exonic and intronic GC contents should be similar [3]. Thus the observed inverse correlations may indicate that natural selection should have played a role in shaping the distinct synonymous codon usage in different tissues. This was confirmed by the observations that CAI values are significantly positively correlated with GC3, but not with intronic GC content in all tested tissues (data not shown). If tissue-specific genes were under selective pressure to optimize translation, it expects that introns would be not influenced by this effect [6]. Notwithstanding, no significant correlation between Axis 1 coordinates and CAI values was found (Spearman’s correlation coefficient *r* = −0.75, *p* = 0.066). Overall, the results indicate that the tissue-specific variability of synonymous codon usage is mainly due to base compositional mutation bias, while translational selection contributes to, with a relative smaller fraction, in shaping this phenomenon. Thus, although rice and human share a similar isochore genomic structure [9], the features of codons that are responsible for the tissue specificity of synonymous codon usage in rice are not totally the same as that in the human genome [6].

### Selective Constraints on Tissue- and Non-tissue-specific Genes

The analyses of tissue-specific genes reveal that the evolution of synonymous codon usage of a given gene may essentially depend on the genomic environment where it is located [6], [32]. Because weak tissue-specific variation of GC3 content exists, I would expect that tissue-specifically expressed genes may be strongly biased to cluster into GC-rich or GC-poor regions along the rice chromosomes. However, like in the human genome, this study did not find any significant clustering of tissue-specific genes along rice chromosomes. Conversely, these genes are distributed throughout the genome (see Fig. S1). Actually, housekeeping but not tissue-specific genes are prone to cluster in chromosomal regions [33]. Moreover, the genes that are selectively expressed in different tissues have near similar distributions of CDS lengths (Table 1). Hence, these factors are not sufficient to explain the tissue-specific variation of GC3 content.

As mentioned above, the variability of synonymous codon usage between tissues may be partly due to translational selection. Thus, the synonymous (*d*_S_) and nonsynonymous substitution patterns (*d*_N_) between rice and *B. distachyon*, and *S. bicolor* were compared to investigate the evolutionary rate of tissue- and non-tissue-specific genes. Here, if a rice gene’s SPM values are less than 0.4 in all tested tissues, it is considered as non-tissue-specific. The final dataset contains 1,187 non-tissue-specific genes in rice. In addition, by searching the PhylomeDB and MetaPhOrs databases, a total of 1,600 and 297 orthologous gene pairs were identified in *B. distachyon* and *S. bicolor*, respectively, in which 431 and 82 genes match well with the rice tissue-specific genes. Further, based on the EST profiling analysis, 60 and 493 genes were finally characterized as the tissue- and non-tissue-specific orthologs in *B. distachyon*. Comparatively, 111 genes consisting of 25 tissue-specific and 86 non-tissue-specific sequences were identified in *S. bicolor*. Of the 60 and 25 tissue-specific genes identified in *B. distachyon* and *S. bicolor*, there are 25 and 7 leaf-specific, and 15 and 8 anther-specific genes in these two species, respectively.

The comparison of *d*_S_ and *d*_N_ rate values of orthologous gene pairs shows that tissue-specific genes are absolutely under weaker selective constraints, as reflected from their significant higher average synonymous (*d*_S_) and nonsynonymous substitution rates (*d*_N_) when compared with non-tissue-specific genes (Fig. 3). From Table 1, it is obvious that anther-specific genes have a higher expression level and a stronger codon usage bias than those selectively expressed in leaf, as reflected from their CAI and ENC values. Further, analysis of *d*_S_ and *d*_N_ rates for the orthologous gene pairs of the two tissues reveals that leaf-specific genes (*d*_N_ = 0.137, and 0.133; *d*_S_ = 0.964, and 1.050, respectively) are under stronger selective constraints than those of anther-specific genes (*d*_N_ = 0.161, and 0.157; *d*_S_ = 1.908, and 1.720, respectively) in both *B. distachyon* and *S. bicolor*. The similar cases were also observed by analyzing the comparison of other tissue genes (data not shown). These results strongly support the conclusion that translational selection is indeed involved in the processes of shaping the tissue-specific synonymous codon usage in the rice genome.

## Supporting Information

Figure S1 **Distribution of tissue-specific genes onto rice chromosomes.** The tissue names were used to represent the genes specifically expressed in that tissue.
(PDF)

Table S1 **EST abundance, CAI values, and functional annotation of 71 highly expressed genes in rice.**
(DOC)

## References

[1] Bulmer M (1991) The selection-mutation-drift theory of synonymous codon usage. Genetics 129: 897–907. 1752426 10.1093/genetics/129.3.897 PMC1204756

[2] Duret L, Mouchiroud D (1999) Expression pattern and, surprisingly, gene length shape codon usage in *Caenorhabditis*, *Drosophila*, and *Arabidopsis* . Proc Natl Acad Sci U S A 96: 4482–4487. 10200288 10.1073/pnas.96.8.4482 PMC16358

[3] Qiu S, Bergero R, Zeng K, Charlesworth D (2011) Patterns of codon usage bias in *Silene latifolia* . Mol Biol Evol 28: 771–780. 20855431 10.1093/molbev/msq251

[4] Duret L (2002) Evolution of synonymous codon usage in metazoans. Curr Opin Genet Dev 12: 640–669. 12433576 10.1016/s0959-437x(02)00353-2

[5] Plotkin JB, Robins H, Levine AJ (2004) Tissue-specific codon usage and the expression of human genes. Proc Natl Acad Sci U S A 101: 12588–12591. 15314228 10.1073/pnas.0404957101 PMC515101

[6] Sémon M, Lobry JR, Duret L (2006) No evidence for tissue-specific adaptation of synonymous codon usage in humans. Mol Biol Evol 23: 523–529. 16280544 10.1093/molbev/msj053

[7] Zhang L, Li WH (2004) Mammalian housekeeping genes evolve more slowly than tissue-specific genes. Mol Biol Evol 21: 236–239. 14595094 10.1093/molbev/msh010

[8] Mukhopadhyay P, Basak S, Ghosh TC (2008) Differential selective constraints shaping codon usage pattern of housekeeping and tissue-specific homologous genes of rice and *Arabidopsis* . DNA Res 15: 347–356. 18827062 10.1093/dnares/dsn023 PMC2608846

[9] Sharp PM, Averof M, Lloyd AT, Matassi G, Peden JF (1995) DNA sequence evolution: the sounds of silence. Philos Trans R Soc Lond Ser B Biol Sci 349: 241–247. 8577834 10.1098/rstb.1995.0108

[10] Liu Q, Feng Y, Zhao X, Dong H, Xue Q (2004) Synonymous codon usage bias in *Oryza sativa* . Plant Sci 167: 101–105.

[11] Cazes P, Chessel D, Doledec S (1988) L’analyse des correspondances internes d’un tableau partitionné: son usage en hydrobiology. Rev Stat Appl 36: 39–54.

[12] Morgulis A, Coulouris G, Raytselis Y, Madden TL, Agarwala R, et al (2008) Database indexing for production MegaBLAST searches. Bioinformatics 24: 1757–1764. 18567917 10.1093/bioinformatics/btn322 PMC2696921

[13] Li M, Xu W, Yang W, Kong Z, Xue Y (2007) Genome-wide gene expression profiling reveals conserved and novel molecular functions of the stigma in rice. Plant Physiol 144: 1797–1812. 17556504 10.1104/pp.107.101600 PMC1949881

[14] Xue LJ, Zhang JJ, Xue HW (2009) Characterization and expression profiles of miRNAs in rice seeds. Nucleic Acids Res 37: 916–930. 19103661 10.1093/nar/gkn998 PMC2647296

[15] Wang YP, Liang L, Han BC, Quan Y, Wang X, et al (2006) GEPS: the gene expression pattern scanner. Nucleic Acids Res 34: W492–W497. 16845057 10.1093/nar/gkl067 PMC1538815

[16] Wright F (1990) The ‘effective number of codons’ used in a gene. Gene 87: 23–29. 2110097 10.1016/0378-1119(90)90491-9

[17] Sharp PM, Li WH (1987) The codon adaptation index – a measure of directional synonymous codon usage bias, and its potential applications. Nucleic Acids Res 15: 1281–1295. 3547335 10.1093/nar/15.3.1281 PMC340524

[18] Perrière G, Thioulouse J (2002) Use and misuse of correspondence analysis in codon usage studies. Nucleic Acids Res 30: 4548–4555. 12384602 10.1093/nar/gkf565 PMC137129

[19] Charif D, Lobry JR (2007) SeqinR 1.0–2: a contributed package to the R project for statistical computing devoted to biological sequences retrieval and analysis. In: Bastolla U, Porto M, Roman HE, Vendruscolo M, editors. Structural approaches to sequence evolution: Molecules, networks, populations. Biological and Medical Physics, Biomedical Engineering. p. 207–232.

[20] Dray S, Dufour AB (2007) The ade4 package: implementing the duality diagram for ecologists. J Stat Softw 22: 1–20.

[21] R Development Core Team (2003) R: a language and environment for statistical computing. Vienna, Austria.

[22] Lobry J, Chessel D (2003) Internal correspondence analysis of codon and amino-acid usage in thermophilic bacteria. J Appl Genet 44: 235–261. 12817570

[23] Peden JF (1999) Analysis of codon usage. PhD Thesis, University of Nottingham, UK. p. 50–90.

[24] Huerta-Cepas J, Capella-Gutierrez S, Pryszcz LP, Denisov I, Kormes D, et al (2011) PhylomeDB v3.0: an expanding repository of genome-wide collections of trees, alignments and phylogeny-based orthology and paralogy predictions. Nucleic Acids Res 39: D556–D560. 21075798 10.1093/nar/gkq1109 PMC3013701

[25] Pryszcz LP, Huerta-Cepas J (2011) Gabaldón (2011) MetaPhOrs: orthology and paralogy predictions from multiple phylogenetics evidence using a consistency-based confidence score. Nucleic Acids Res 39: e32. 21149260 10.1093/nar/gkq953 PMC3061081

[26] Dessimoz C, Gabaldón T, Roos DS, Sonnhammer EL, Herrero J, et al (2012) Toward community standards in the quest for orthologs. Bioinformatics 28: 900–904. 22332236 10.1093/bioinformatics/bts050 PMC3307119

[27] Koski LB, Golding GB (2001) The closest BLAST hit is often not the nearest neighbor. J Mol Evol 52: 540–542. 11443357 10.1007/s002390010184

[28] Katoh K, Toh H (2010) Parallelization of the MAFFT multiple sequence alignment program. Bioinformatics 26: 1899–1900. 20427515 10.1093/bioinformatics/btq224 PMC2905546

[29] Yang Z, Nielsen R (2000) Estimating synonymous and nonsynonymous substitution rates under realistic evolutionary models. Mol Biol Evol 17: 32–43. 10666704 10.1093/oxfordjournals.molbev.a026236

[30] Yang Z (2007) PAML 4: phylogenetic analysis by maximum likelihood. Mol Biol Evol 24: 1586–1591. 17483113 10.1093/molbev/msm088

[31] Milnthorpe AT, Soloviev M (2012) The use of EST expression matrixes for the quality control of gene expression data. PLoS One 7: e32966. 22412959 10.1371/journal.pone.0032966 PMC3297614

[32] Vinogradov AE (2003) Isochores and tissue-specificity. Nucleic Acids Res 31: 5212–5220. 12930973 10.1093/nar/gkg699 PMC212799

[33] Lercher MJ, Urrutia AO, Hurst LD (2002) Clustering of housekeeping genes provides a unified model of gene order in the human genome. Nat Genet 31: 180–183. 11992122 10.1038/ng887

