# Supplementary material for: Mutational Bias and Translational Selection Shaping the Codon Usage Pattern of Tissue-Specific Genes in Rice
Source: PLoS One. 2012 Oct 29;7(10):e48295. doi: 10.1371/journal.pone.0048295 (PMC3483185; doi:10.1371/journal.pone.0048295)
Supplement: Figure S1 — Distribution of tissue-specific genes onto rice chromosomes. The tissue names were used to represent the genes specifically expressed in that tissue. (PDF) [file pone.0048295.s001.pdf]

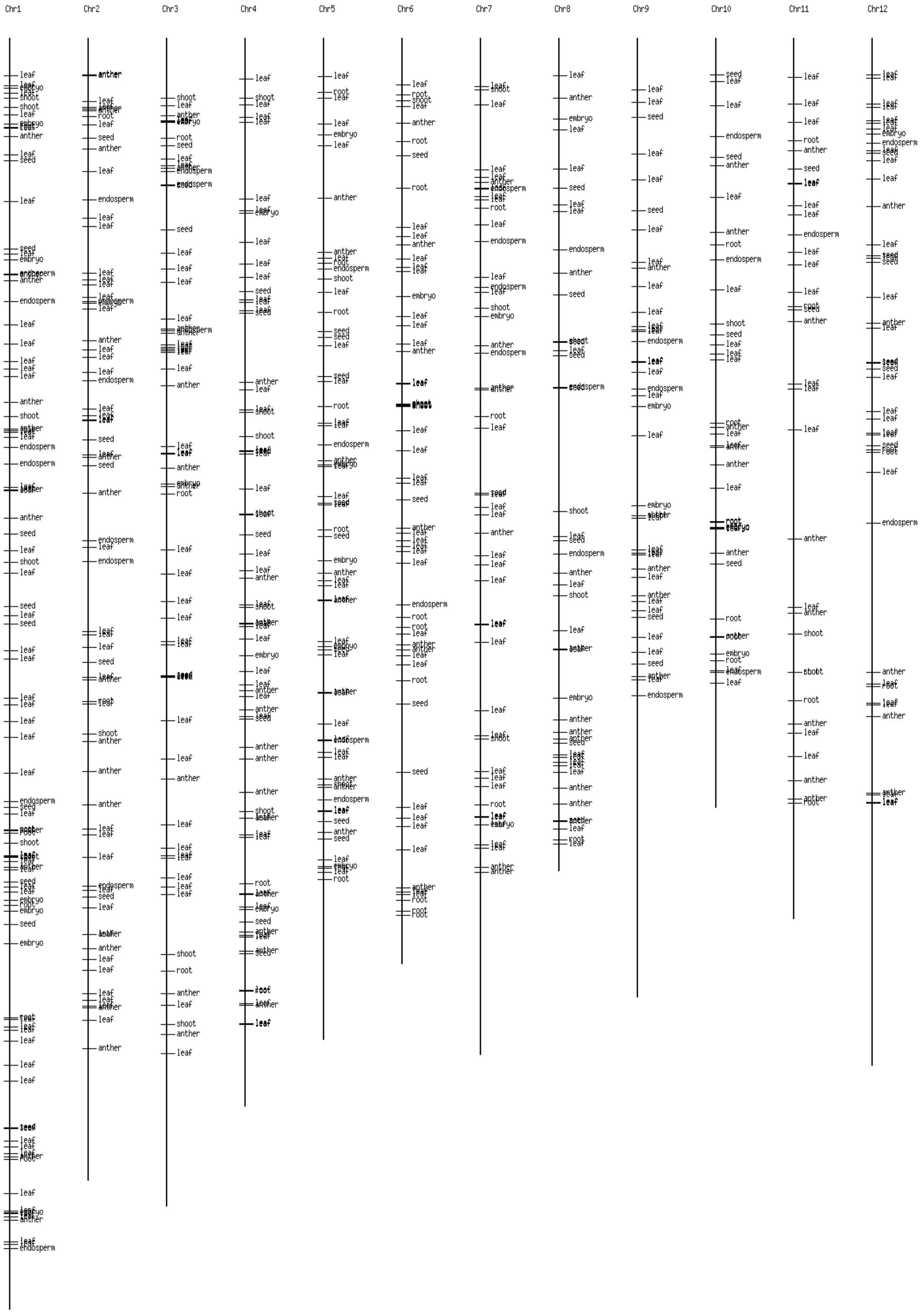

Supplemental Figure 1

Distribution of tissue-specific genes onto rice chromosomes. The tissue names were used to represent the genes specifically expressed in that tissue.
