# Supplementary material for: Mutational Bias and Translational Selection Shaping the Codon Usage Pattern of Tissue-Specific Genes in Rice
Source: PLoS One. 2012 Oct 29;7(10):e48295. doi: 10.1371/journal.pone.0048295 (PMC3483185; doi:10.1371/journal.pone.0048295)
Supplement: Table S1 — EST abundance, CAI values, and functional annotation of 71 highly expressed genes in rice. (DOC) [file pone.0048295.s002.doc]

Table S1. EST abundance, CAI values, and functional annotation of 71 highly expressed genes in rice.

| Gene ID | EST counts | CAI value | Functional annotation |
| --- | --- | --- | --- |
| LOC_Os01g03340.1 | 532 | 0.858 | BBTI4 - Bowman-Birk type bran trypsin inhibitor precursor, expressed |
| LOC_Os01g03360.1 | 507 | 0.834 | BBTI5 - Bowman-Birk type bran trypsin inhibitor precursor, expressed |
| LOC_Os01g03950.1 | 314 | 0.797 | glycosyl hydrolase, family 31, putative, expressed |
| LOC_Os01g18050.1 | 446 | 0.806 | tubulin/FtsZ domain containing protein, putative, expressed |
| LOC_Os01g31690.1 | 658 | 0.832 | oxygen-evolving enhancer protein 1, chloroplast precursor, putative, expressed |
| LOC_Os01g41710.1 | 2248 | 0.913 | chlorophyll A-B binding protein, putative, expressed |
| LOC_Os01g52240.1 | 1901 | 0.894 | chlorophyll A-B binding protein, putative, expressed |
| LOC_Os01g62290.1 | 573 | 0.863 | DnaK family protein, putative, expressed |
| LOC_Os01g67860.1 | 326 | 0.784 | fructose-bisphospate aldolase isozyme, putative, expressed |
| LOC_Os01g74450.1 | 453 | 0.862 | aquaporin protein, putative, expressed |
| LOC_Os02g02490.1 | 470 | 0.800 | phytosulfokine receptor precursor, putative, expressed |
| LOC_Os02g06640.1 | 4085 | 0.803 | ubiquitin family protein, putative, expressed |
| LOC_Os02g10390.1 | 450 | 0.811 | chlorophyll A-B binding protein, putative, expressed |
| LOC_Os02g44630.1 | 721 | 0.752 | aquaporin protein, putative, expressed |
| LOC_Os02g52700.1 | 301 | 0.843 | alpha-amylase precursor, putative, expressed |
| LOC_Os02g53860.1 | 525 | 0.805 | OsSub22 - Putative Subtilisin homologue, expressed |
| LOC_Os03g01530.1 | 331 | 0.916 | tubulin/FtsZ domain containing protein, putative, expressed |
| LOC_Os03g05290.1 | 568 | 0.915 | aquaporin protein, putative, expressed |
| LOC_Os03g08010.1 | 1706 | 0.767 | elongation factor Tu, putative |
| LOC_Os03g08020.1 | 1707 | 0.767 | elongation factor Tu, putative, expressed |
| LOC_Os03g08050.1 | 1707 | 0.755 | elongation factor Tu, putative, expressed |
| LOC_Os03g08060.1 | 1707 | 0.756 | elongation factor Tu, putative, expressed |
| LOC_Os03g08360.1 | 321 | 0.880 | 3-ketoacyl-CoA synthase 10, putative, expressed |
| LOC_Os03g16860.1 | 1279 | 0.882 | DnaK family protein, putative, expressed |
| LOC_Os03g16920.1 | 501 | 0.892 | DnaK family protein, putative, expressed |
| LOC_Os03g20780.1 | 348 | 0.814 | ethylene-insensitive 3, putative |
| LOC_Os03g20790.1 | 348 | 0.820 | ethylene-insensitive 3, putative |
| LOC_Os03g39610.1 | 1558 | 0.855 | chlorophyll A-B binding protein, putative, expressed |
| LOC_Os03g45920.1 | 354 | 0.884 | tubulin/FtsZ domain containing protein, putative, expressed |
| LOC_Os03g46100.1 | 511 | 0.808 | cupin domain containing protein, expressed |
| LOC_Os03g51600.1 | 1055 | 0.762 | tubulin/FtsZ domain containing protein, putative, expressed |
| LOC_Os03g53860.1 | 332 | 0.818 | periplasmic beta-glucosidase precursor, putative, expressed |
| LOC_Os04g01740.1 | 693 | 0.897 | heat shock protein, putative, expressed |
| LOC_Os04g16450.1 | 348 | 0.770 | aquaporin protein, putative, expressed |
| LOC_Os04g38600.1 | 397 | 0.865 | glyceraldehyde-3-phosphate dehydrogenase, putative, expressed |
| LOC_Os04g43760.1 | 737 | 0.889 | phenylalanine ammonia-lyase, putative, expressed |
| LOC_Os04g47220.1 | 596 | 0.799 | aquaporin protein, putative, expressed |
| LOC_Os04g53620.1 | 1018 | 0.758 | ubiquitin family protein, putative, expressed |
| LOC_Os05g12400.1 | 2119 | 0.758 | BURP domain containing protein, expressed |
| LOC_Os05g12410.1 | 2170 | 0.856 | BURP domain containing protein |
| LOC_Os05g12640.1 | 1763 | 0.827 | BURP domain containing protein, expressed |
| LOC_Os05g35290.1 | 356 | 0.882 | phenylalanine ammonia-lyase, putative, expressed |
| LOC_Os05g38530.1 | 1282 | 0.898 | DnaK family protein, putative, expressed |
| LOC_Os05g44340.1 | 354 | 0.836 | heat shock protein 101, putative, expressed |
| LOC_Os05g44770.1 | 331 | 0.768 | receptor-like protein kinase 5 precursor, putative, expressed |
| LOC_Os06g07220.1 | 461 | 0.826 | LTPL128 - Protease inhibitor/seed storage/LTP family protein precursor, expressed |
| LOC_Os06g46284.1 | 1361 | 0.833 | glycosyl hydrolase, family 31, putative, expressed |
| LOC_Os06g46340.1 | 1365 | 0.808 | glycosyl hydrolase, family 31, putative, expressed |
| LOC_Os06g46770.1 | 5575 | 0.839 | ubiquitin family protein, putative, expressed |
| LOC_Os07g26690.1 | 362 | 0.878 | aquaporin protein, putative, expressed |
| LOC_Os07g37550.1 | 2114 | 0.897 | chlorophyll A-B binding protein, putative, expressed |
| LOC_Os07g38960.1 | 508 | 0.865 | chlorophyll A-B binding protein, putative, expressed |
| LOC_Os07g48460.1 | 542 | 0.845 | stress responsive protein, putative, expressed |
| LOC_Os07g48500.1 | 419 | 0.852 | stress responsive protein, putative, expressed |
| LOC_Os08g35760.1 | 314 | 0.880 | Cupin domain containing protein, expressed |
| LOC_Os08g36900.1 | 335 | 0.843 | alpha-amylase precursor, putative, expressed |
| LOC_Os08g36910.1 | 489 | 0.806 | alpha-amylase precursor, putative, expressed |
| LOC_Os08g39140.1 | 1690 | 0.769 | heat shock protein, putative, expressed |
| LOC_Os09g17740.1 | 2174 | 0.905 | chlorophyll A-B binding protein, putative, expressed |
| LOC_Os09g25490.1 | 606 | 0.849 | CESA9 - cellulose synthase, expressed |
| LOC_Os09g30412.1 | 1883 | 0.750 | heat shock protein, putative, expressed |
| LOC_Os09g30418.1 | 1963 | 0.763 | heat shock protein, putative |
| LOC_Os09g30439.1 | 1964 | 0.763 | heat shock protein, putative |
| LOC_Os10g32980.1 | 607 | 0.852 | CESA7 - cellulose synthase, expressed |
| LOC_Os11g13890.1 | 316 | 0.862 | chlorophyll A-B binding protein, putative, expressed |
| LOC_Os11g14220.1 | 1127 | 0.811 | tubulin/FtsZ domain containing protein, putative, expressed |
| LOC_Os11g33120.1 | 381 | 0.816 | respiratory burst oxidase, putative, expressed |
| LOC_Os11g47760.1 | 1290 | 0.758 | DnaK family protein, putative, expressed |
| LOC_Os11g47970.1 | 471 | 0.798 | AAA-type ATPase family protein, putative, expressed |
| LOC_Os12g17600.1 | 391 | 0.873 | ribulose bisphosphate carboxylase small chain, chloroplast precursor, putative, expressed |
| LOC_Os12g19381.1 | 366 | 0.789 | ribulose bisphosphate carboxylase small chain, chloroplast precursor, putative, expressed |
